# Supplementary material for: Bakkenolides and Caffeoylquinic Acids from the Aerial Portion of Petasites japonicus and Their Bacterial Neuraminidase Inhibition Ability
Source: Biomolecules. 2020 Jun 10;10(6):888. doi: 10.3390/biom10060888 (PMC7357027; doi:10.3390/biom10060888)
Supplement: Supplementary file 1 [file biomolecules-10-00888-s001.pdf]

Bakkenolides and Caffeoylquinic Acids from the Aerial Portion of *Petasites japonicus* and Their Bacterial Neuraminidase Inhibition Ability

Hyun Sim Woo [1], Kyung-Chul Shin [2], Jeong Yoon Kim [3], Yeong-Su Kim [1], Young Jun Ban [3], Yu Jin Oh [1], Hae Jin Cho [1], Deok-Kun Oh [2] and Dae Wook Kim [1,*]

[1] Plant Resource Industry Division, Forest Plant Industry Department, Baekdudaegan National Arboretum, Bonghwa-gun 26209, Korea; whs0428@bdna.or.kr (H.S.W.); yskim@bdna.or.kr (Y.-S.K.); oyj0705@bdna.or.kr (Y.J.O.); hjlife@bdna.or.kr (H.J.C.)

[2] Department of Bioscience and Biotechnology, Konkuk University, Gwangjin-gu, Seoul 05029, Korea; hidex2@naver.com (K.-C.S.); deokkun@konkuk.ac.kr (D.-K.O.)

[3] Division of Applied Life Science, Gyeongsang National University, Jinju 52828, Korea; yoon24@gnu.ac.kr (J.Y.K.); banyoung972@naver.com (Y.J.B.)

* Correspondence: dwking@bdna.or.kr; Tel.: +82-54-679-2738; Fax: +82-54-679

▣ Characterization Data

Figure S1–20: 1D and 2D NMR spectra of compounds **1**–**4**

Figure S21-22: Isolation of bioactive compounds from the aerial portion of *P. japonicas* with preparative HPLC

Figure S23: HPLC profiles of extract and compounds present in the aerial portion of *P. japonicus*

Figure S24–25: Molecular docking study of inhibition of NA by inhibitors

Table S1: Molecular docking study of inhibition of NA by inhibitors

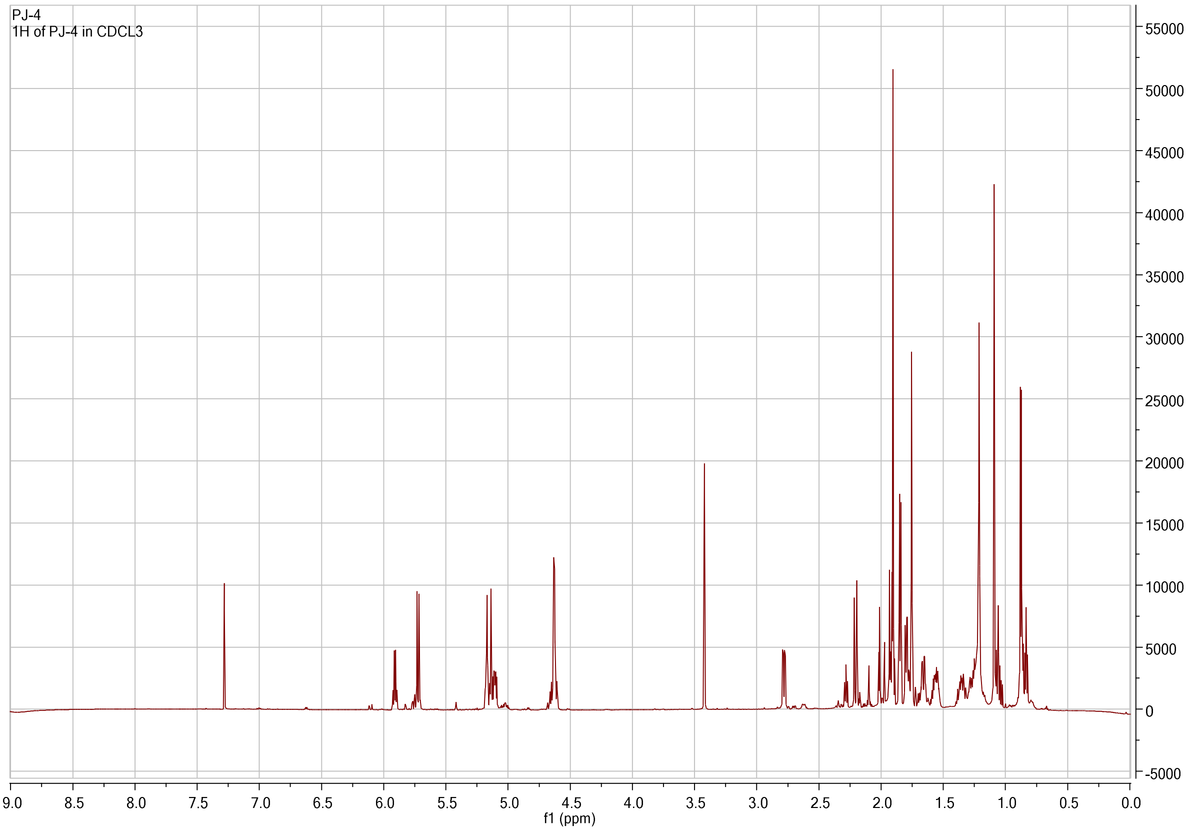

Figure S1. ^{1}H-NMR spectrum of compound **1** ($CDCl_3$ 700 MHz).

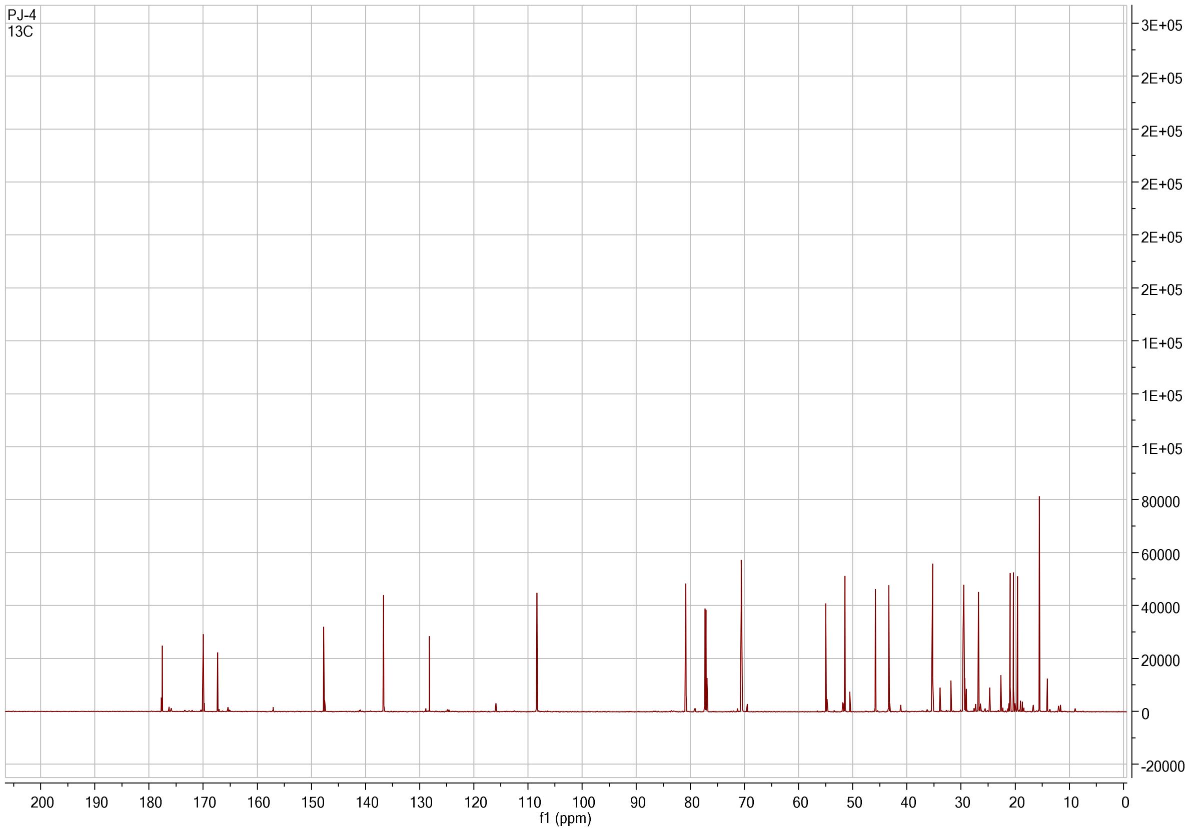

Figure S2. ^{13}C-NMR spectrum of compound **1** ($CDCl_3$ 175 MHz).

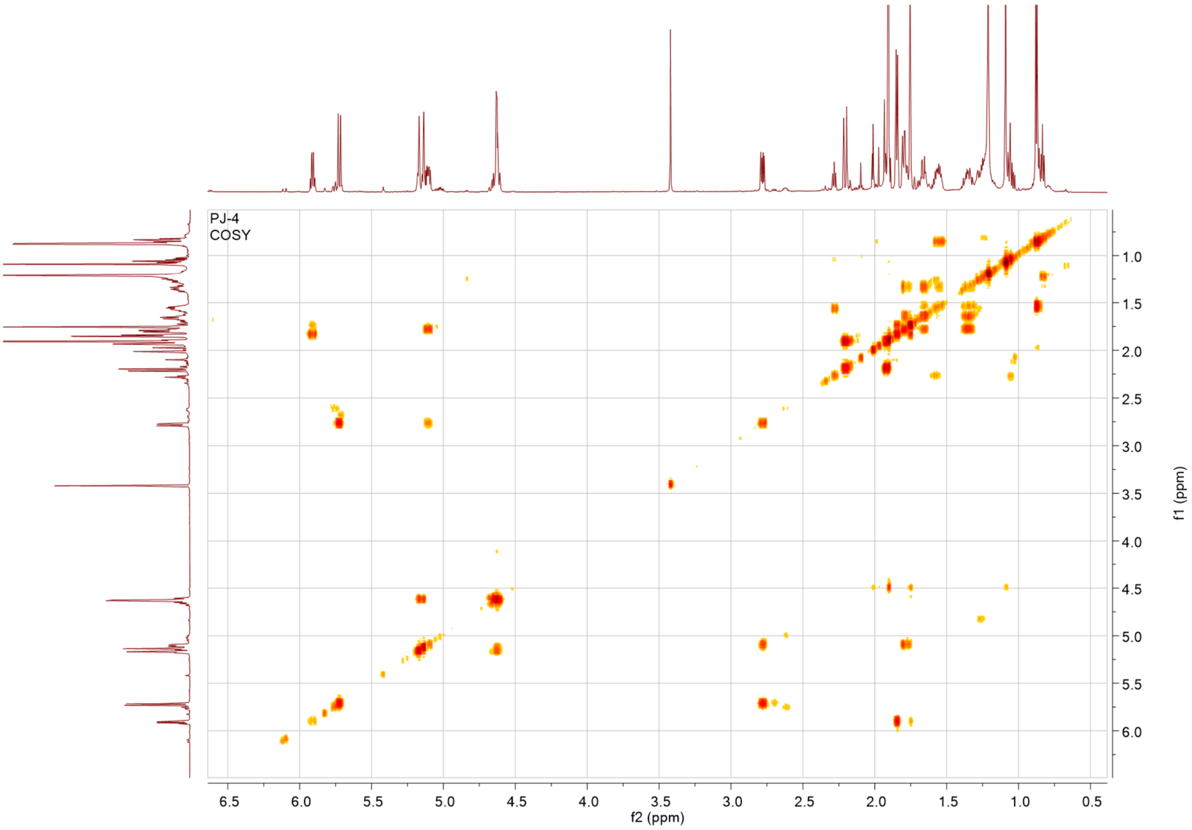

Figure S3. ^{1}H-^{1}H COSY spectrum of compound **1**.

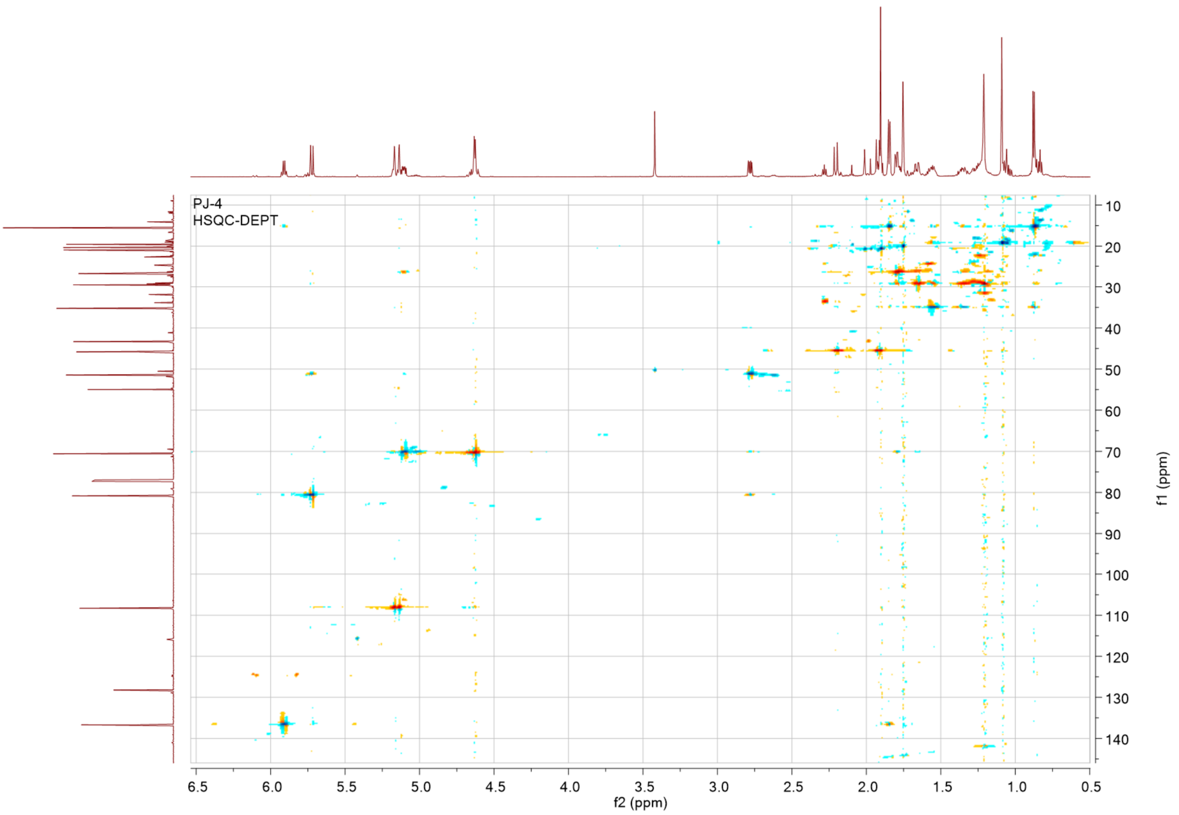

Figure 4. HSQC-DEPT spectrum of compound **1**.

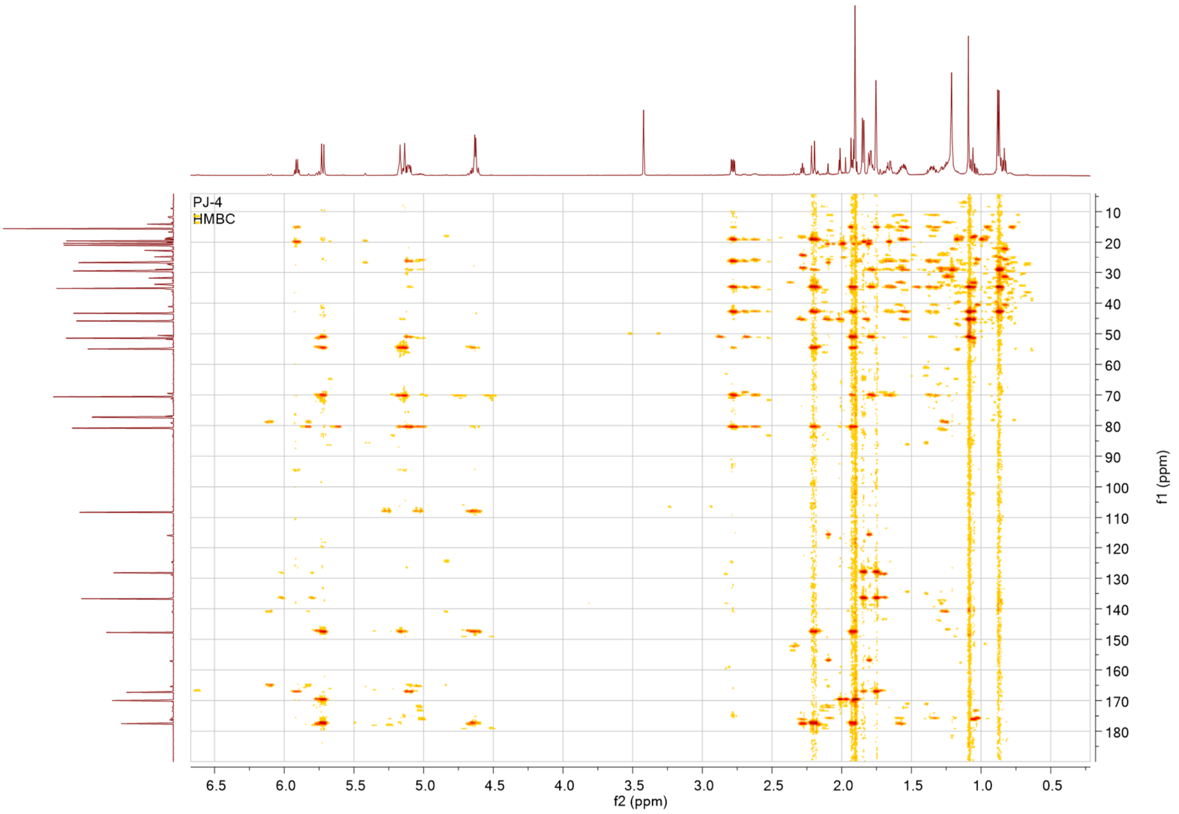

Figure S5. HMBC spectrum of compound **1**.

*Bakkenolide B (**1**)*

^{1}H-NMR (700 MHz, Chloroform-*d*) 5.91 (1H, dd, *J* = 7.2, 15 Hz, H-3′), 5.72 (1H, d, *J* = 11.2 Hz, H-9), 5.17 (1H, s, H-13a), 5.14 (1H, s, H-13b), 5.10 (1H, m, H-1), 4.63 (2H, m, H-12), 2.78 (1H, dd, *J* = 11.2, 5.0 Hz, H-10), 2.21 (1H, d, *J* = 14.3 Hz, H-6), 1.91 (1H, d, *J* = 14.3 Hz, H-6), 1.91 (3H, s, H-2″), 1.85 (3H, dd, *J* = 7.2, 1.6 Hz, H-4′), 1.78 (2H, m, H-2), 1.75 (3H, s, H-5′), 1.66 (1H, m, H-3), 1.55 (1H, m, H-4), 1.34 (1H, m, H-3), 1.09 (3H, s, H-15), 0.87 (3H, d, *J* = 6.8 Hz, H-14). ^{13}C-NMR (175 MHz, Chloroform-*d*) 177.5 (C-8), 169.9 (C-1″), 167.3 (C-1′), 147.7 (C-11), 136.7 (C-3′), 128.2 (C-2′), 108.3 (C-13), 80.8 (C-9), 70.6 (C-12), 70.5 (C-1), 54.9 (C-7), 51.4 (C-10), 45.8 (C-6), 43.4 (C-5), 35.2 (C-4), 29.5 (C-3), 26.8 (C-2), 20.9 (C-2″), 20.3 (C-5′), 19.5 (C-15), 15.5 (C-14), 15.5 (C-4′).

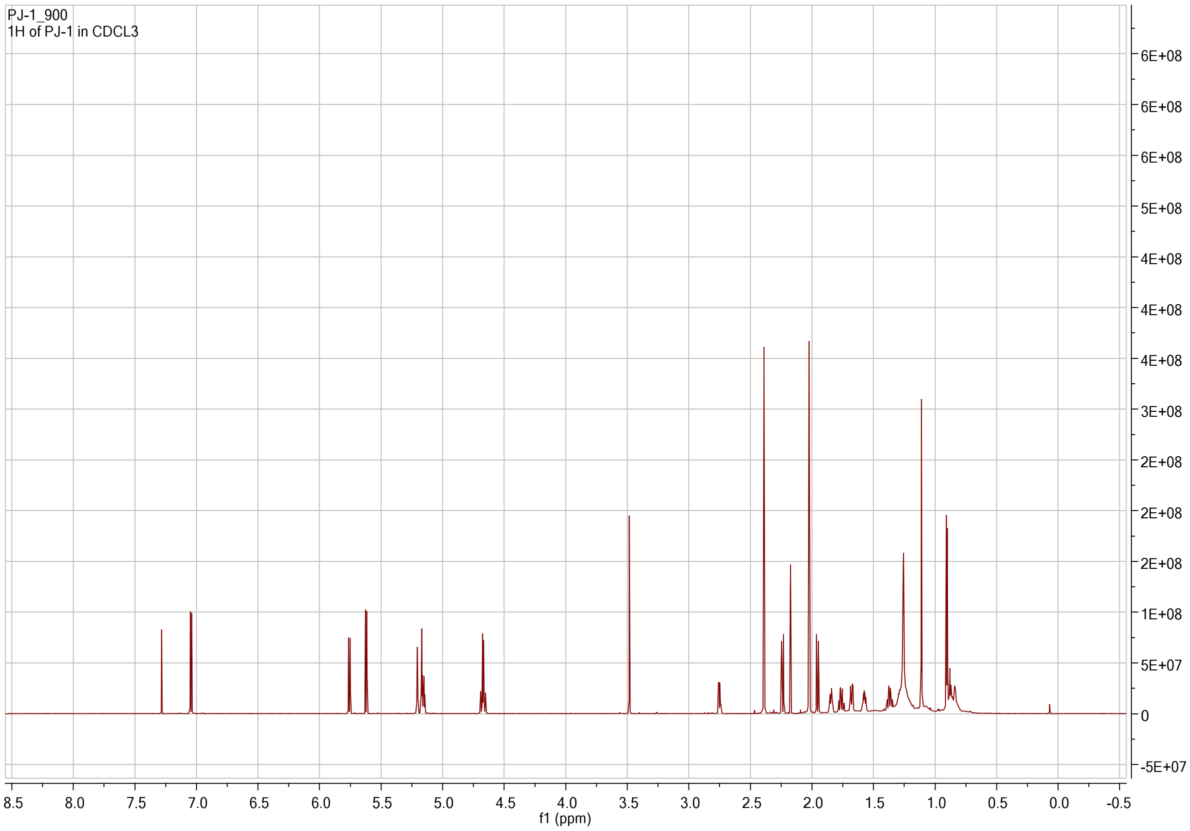

Figure S6. ^{1}H-NMR spectrum of compound **2** ($CDCl_3$ 900 MHz).

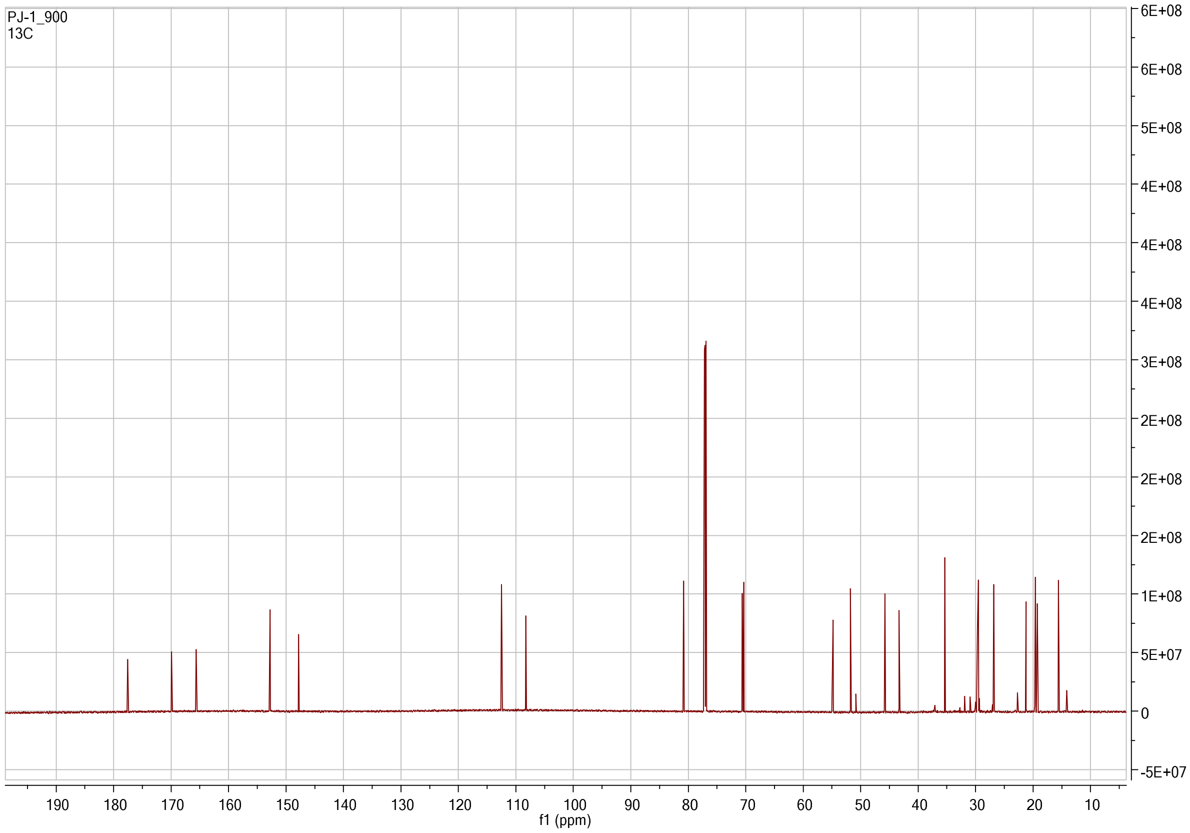

Figure S7. ^{13}C-NMR spectrum of compound **2** ($CDCl_3$ 225 MHz).

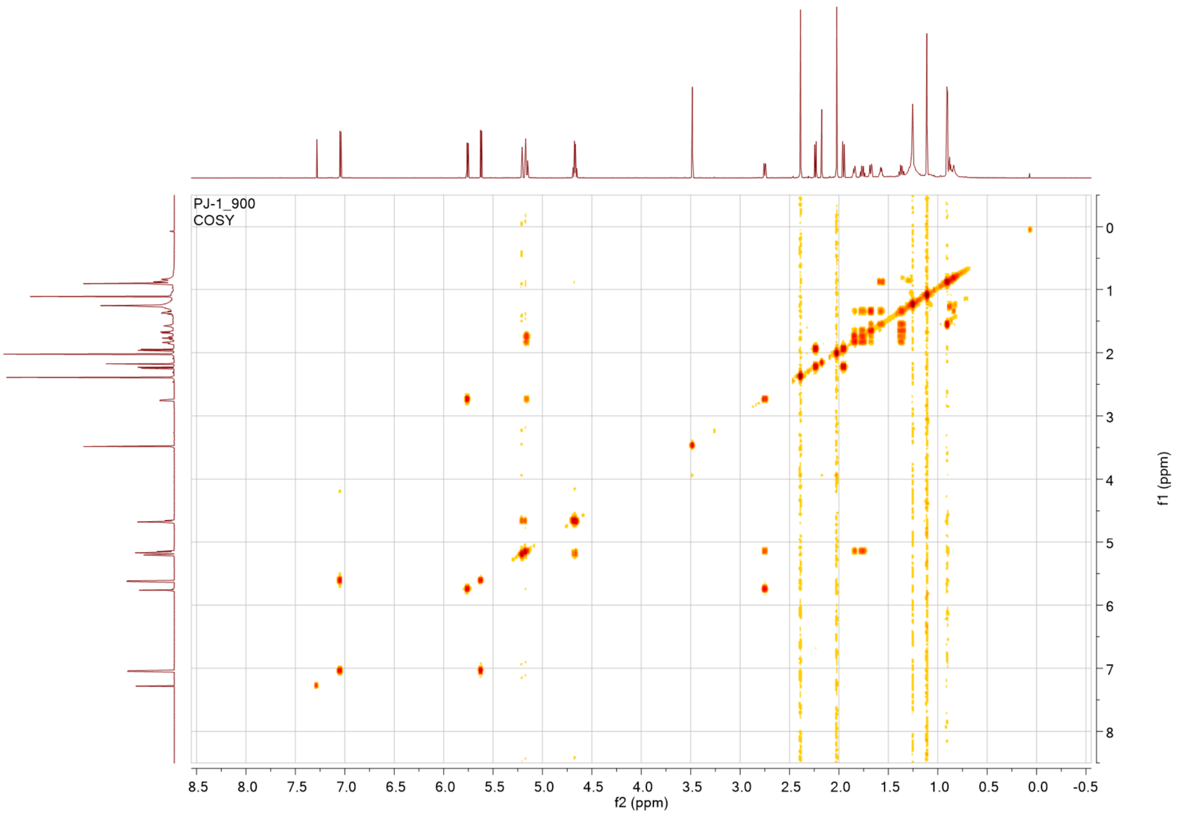

Figure S8. ¹H-¹H COSY spectrum of compound **2**.

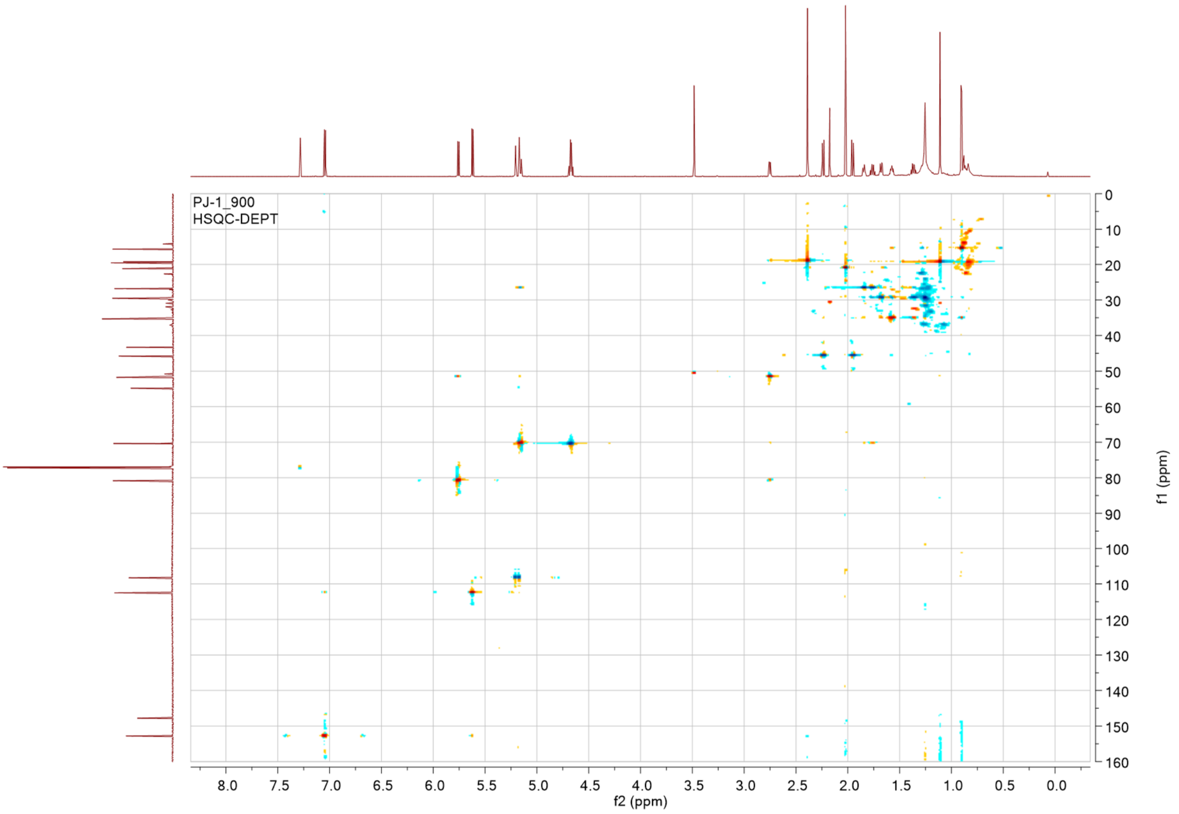

Figure S9. HSQC-DEPT spectrum of compound **2**.

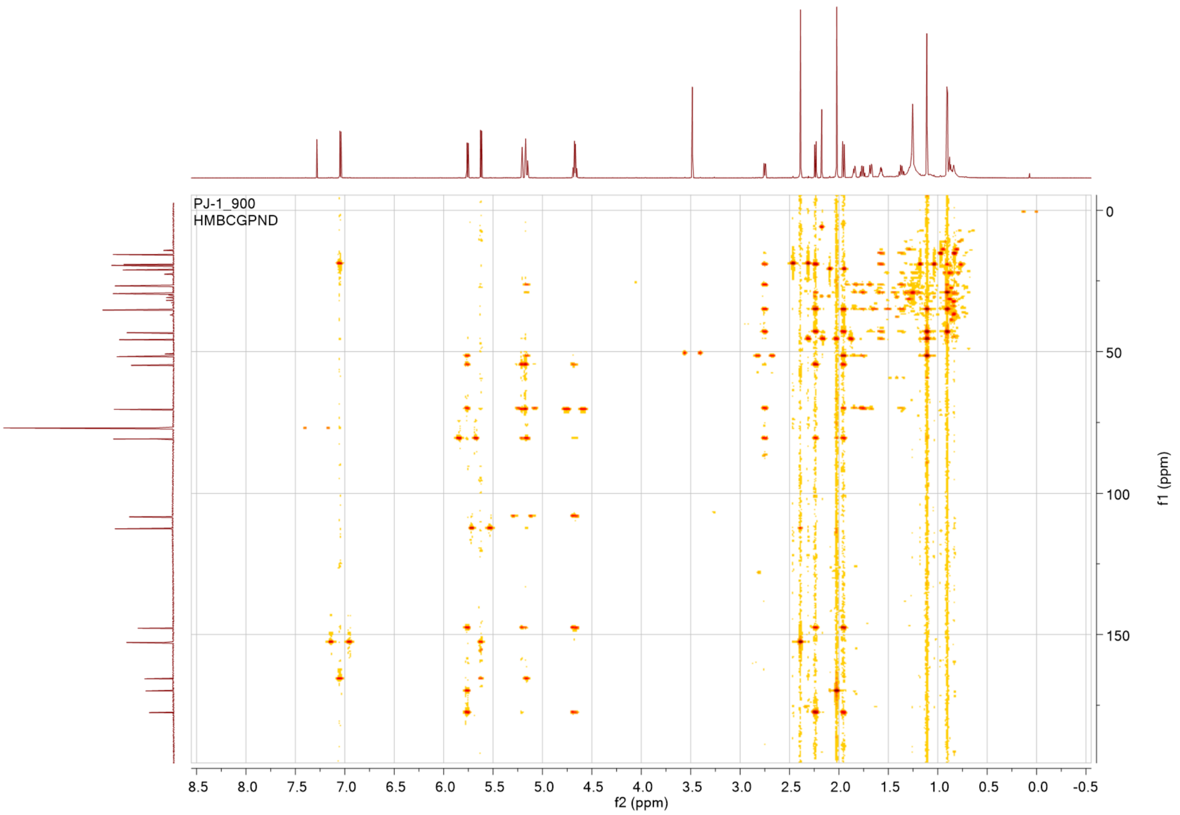

Figure S10. HMBC spectrum of compound **2**.

Bakkenolide D (**2**)

^{1}H-NMR (900 MHz, Chloroform-*d*) 7.04 (1H, d, *J* = 10.1 Hz, H-3′), 5.76 (1H, d, *J* = 11.2 Hz, H-9), 5.62 (1H, d, *J* = 10.14 Hz, H-2′), 5.21 (1H, s, H-13), 5.17 (1H, s, H-13), 5.15 (1H, m, H-1), 4.67 (2H, m, H-12), 2.75 (1H, dd, *J* = 11.2, 5.0 Hz, H-10), 2.39 (3H, s, H-4′), 2.24 (1H, d, *J* = 14.3 Hz, H-6), 2.02 (3H, s, H-2″), 1.95 (1H, d, *J* = 14.3 Hz, H-6), 1.84 (1H, m, H-2), 1.76 (1H, m, H-2), 1.67 (1H, dd, *J* = 14.1, 3.6 Hz, H-3), 1.57 (1H, m, H-4), 1.37 (1H, dd, *J* = 12.9, 3.7 Hz), H-3), 1.11 (3H, s, H-15), 0.90 (3H, d, H-14). ^{13}C-NMR (225 MHz, Chloroform-*d*) 177.5 (C-8), 169.9 (C-1″), 165.6 (C-1′), 152.8 (C-3′), 147.8 (C-11), 112.4 (C-2′), 108.2 (C-13), 80.8 (C-9), 70.5 (C-12), 70.3 (C-1), 54.9 (C-7), 51.7 (C-10), 45.8 (C-6), 43.3 (C-5), 35.3 (C-4), 29.5 (C-3), 26.8 (C-2), 21.2 (C-2″), 19.5 (C-15), 19.2 (C-4′), 15.5 (C-14).

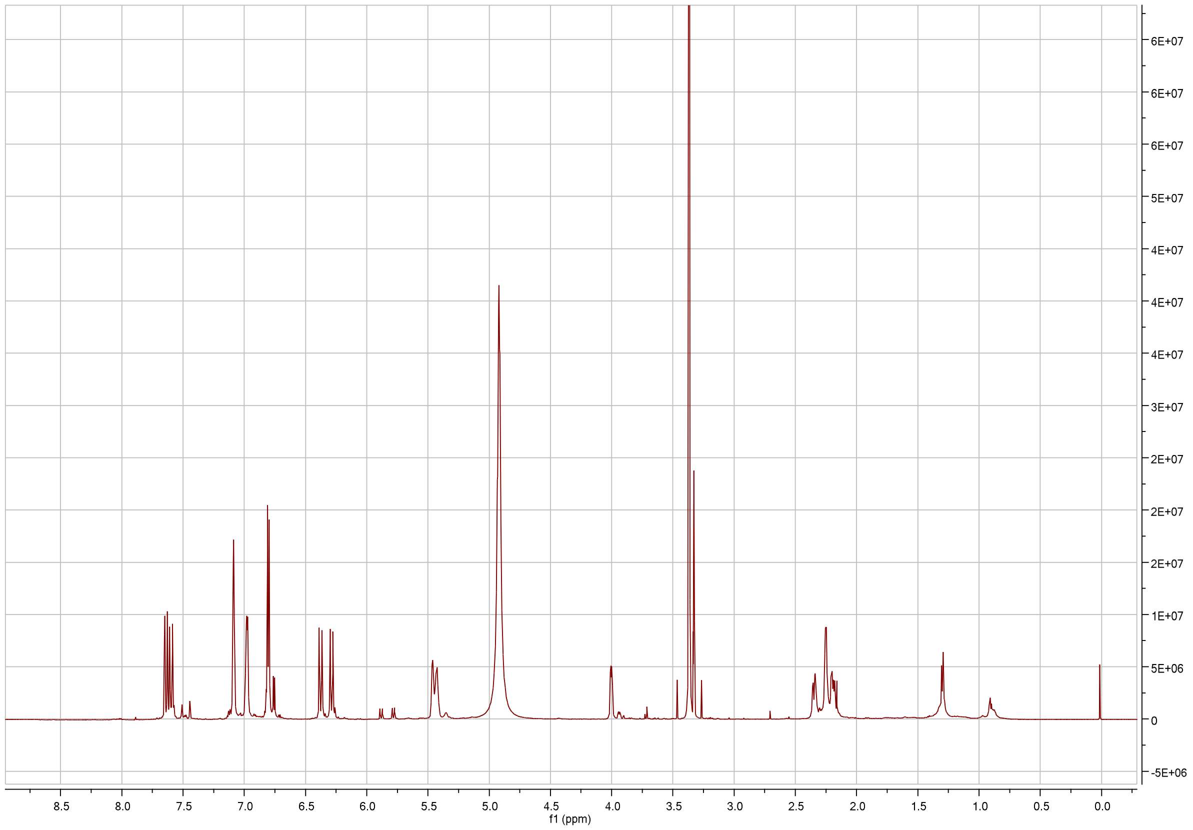

Figure S11. ^{1}H-NMR spectrum of compound **3** (MeOD 700 MHz)

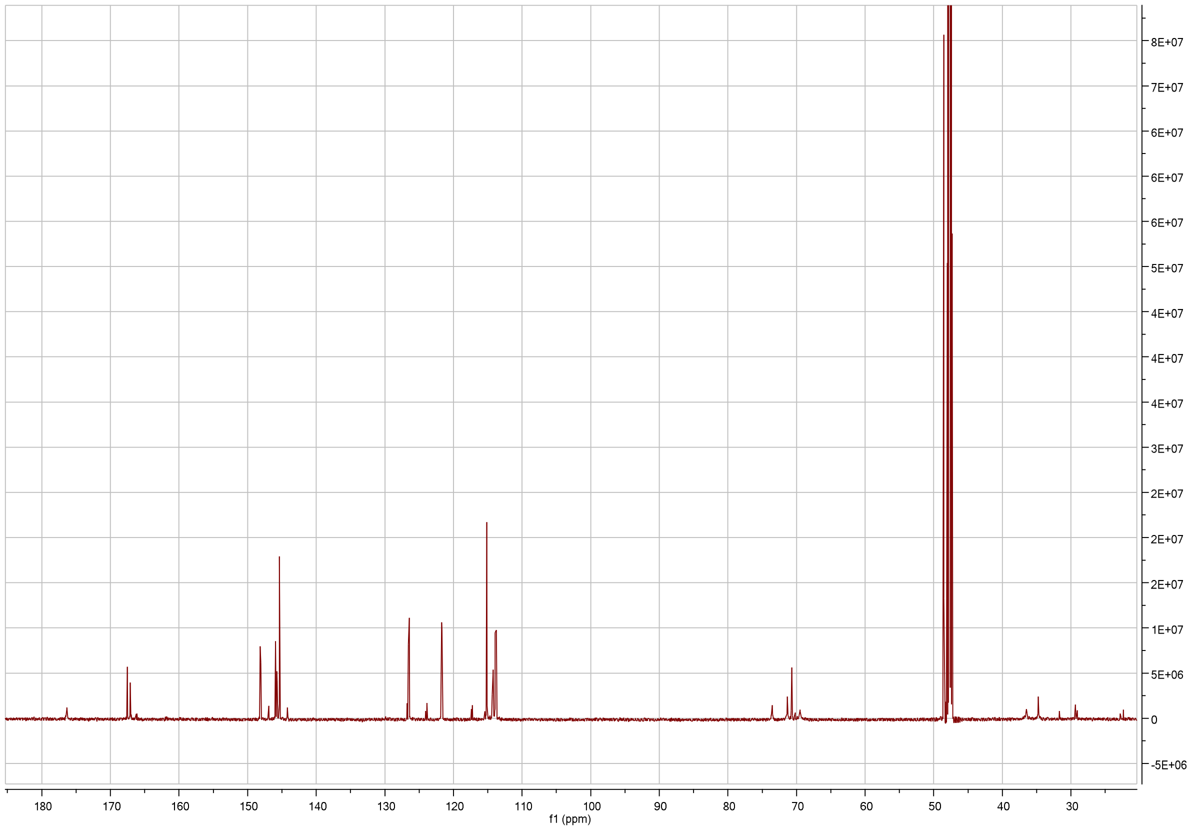

Figure S12. ^{13}C-NMR spectrum of compound **3** (MeOD 175 MHz).

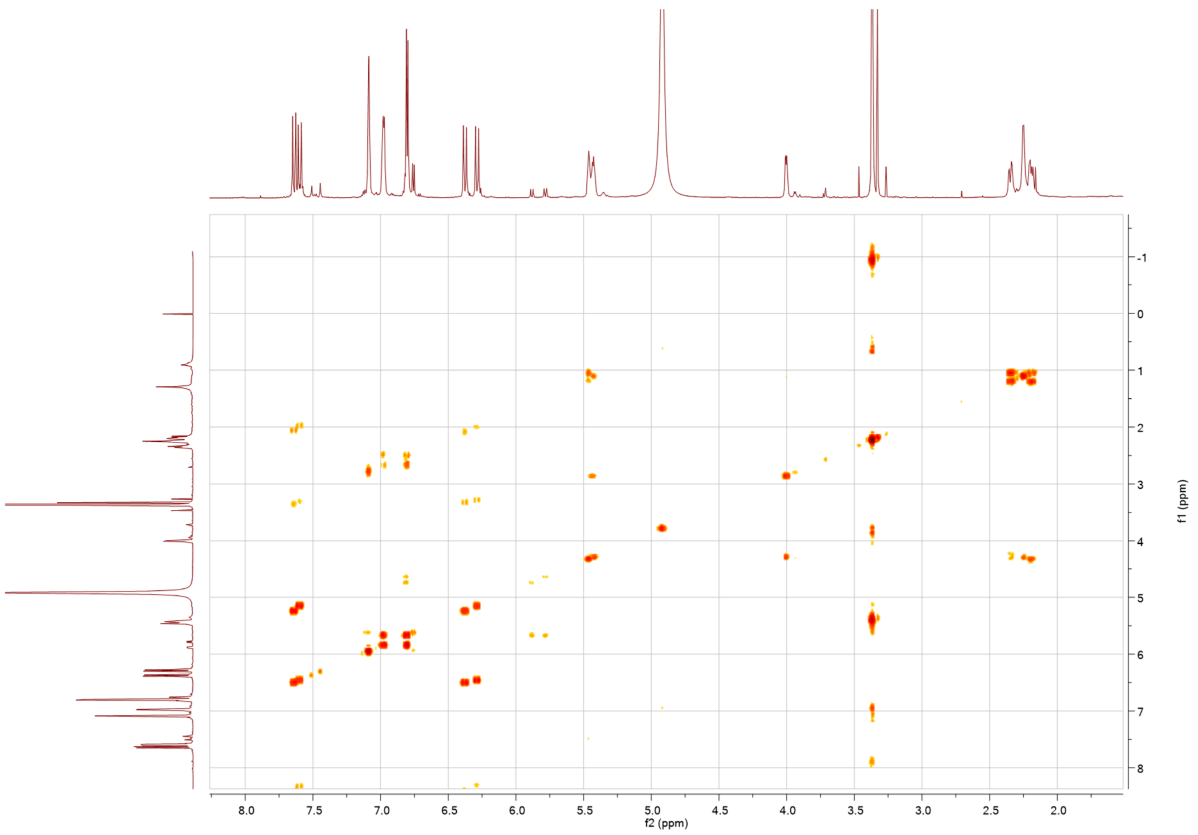

Figure S13. ^1H-^1H COSY spectrum of compound **3**.

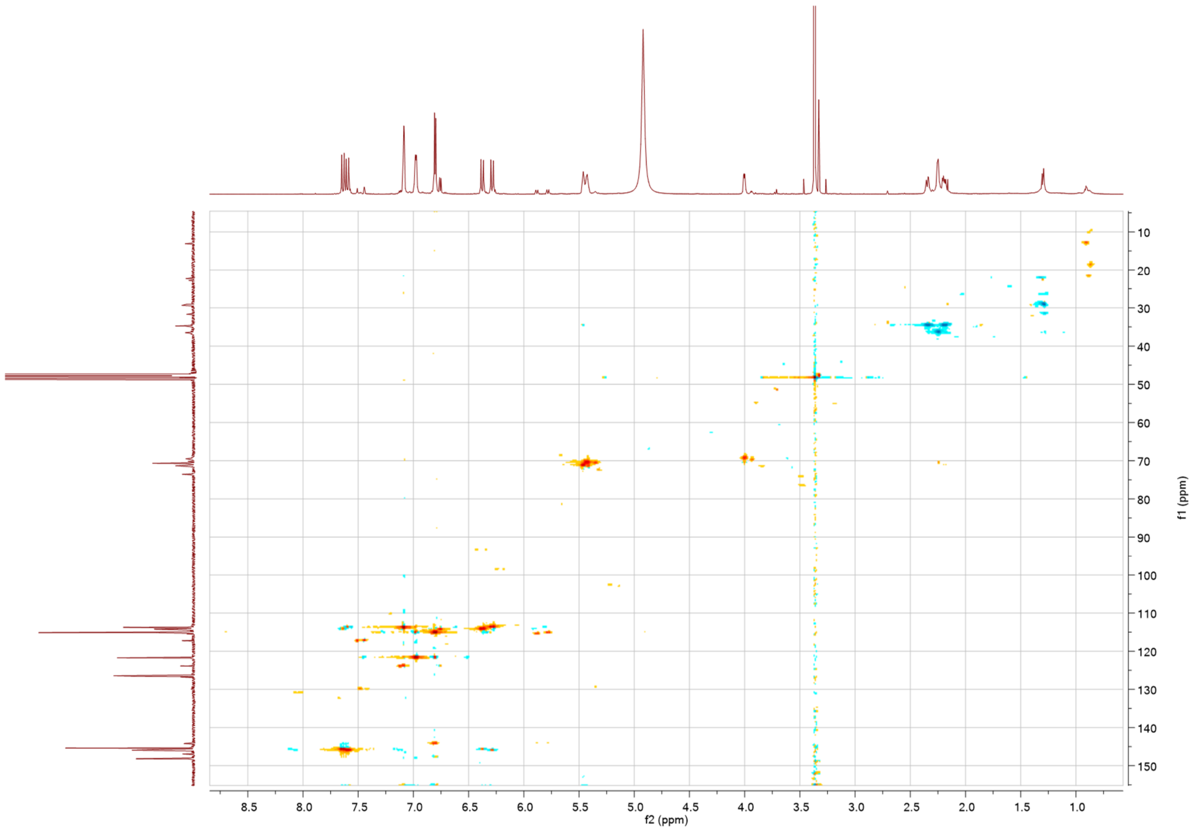

Figure S14. HSQC-DEPT spectrum of compound **3**.

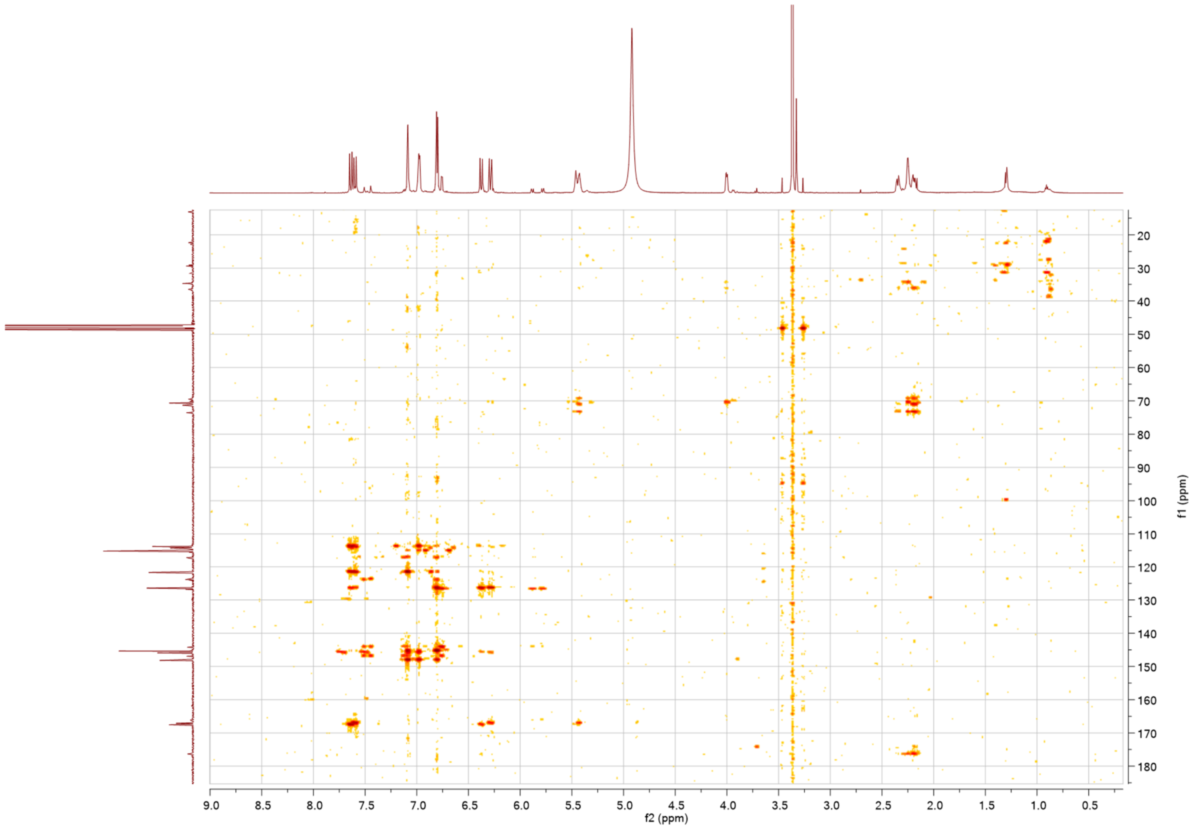

Figure S15. HMBC spectrum of compound **3**.

*1,5-Di-O-caffeoylquinic acid (**3**)*

^{1}H-NMR (700 MHz, Methanol-*d*4) 7.64 (1H, d, *J* = 15.9 Hz, H-7′), 7.60 (1H, d, *J* = 15.9 Hz, H-7″), 7.08 (2H, s, H-2′,2″), 6.97 (2H, m, H-6′,6″), 6.81 (1H, s, H-5′), 6.79 (1H, s, H-5″), 6.38 (1H, d, *J* = 15.9 Hz, H-8′), 6.28 (1H, d, *J* = 15.9 Hz H-8″), 5.46 (1H, m, H-3), 5.42 (1H, m, H-5), 4.00 (1H, dd, *J* = 7.4, 2.8 Hz, H-4), 2.35 (1H, dd, *J* = 13.8, 3.0 Hz, H-6), 2.25 (2H, m, H-2), 2.19 (1H, m, H-6). ^{13}C-NMR (175 MHz, Methanol-*d*4) 176.3 (C-7), 167.5 (C-9′), 167.0 (C-9″), 148.1 (C-4′), 148.0 (C-4″), 145.9 (C-7′), 145.7 (C-7″), 145.3 (C-3′,3″), 126.5 (C-1′), 126.4 (C-1″), 121.7 (C-6′,6″), 115.1 (C-5′,5″), 114.2 (C-8′), 113.9 (C-2′,2″), 113.7 (C-8″), 73.5 (C-1), 71.3 (C-3), 70.6 (C-5), 69.4 (C-4), 36.4 (C-2), 34.7 (C-6).

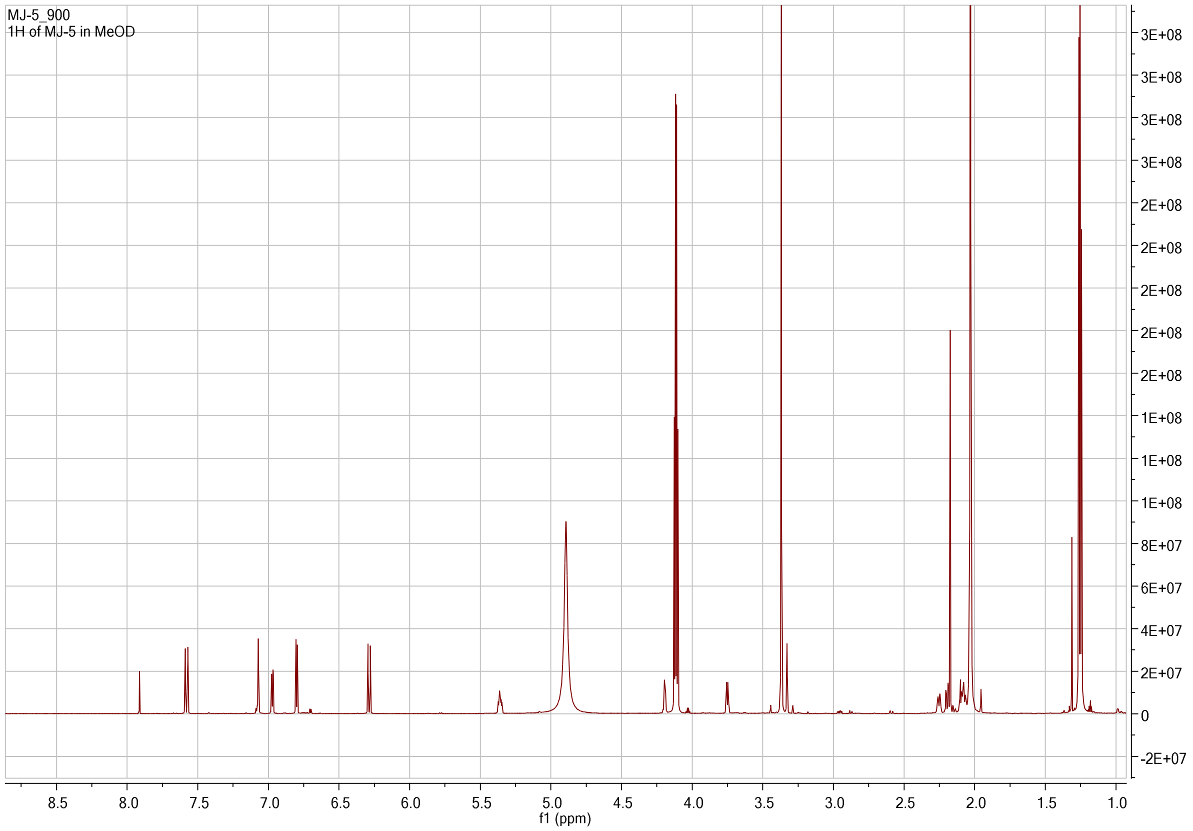

Figure S16. ^{1}H-NMR spectrum of compound **4** (MeOD 900 MHz).

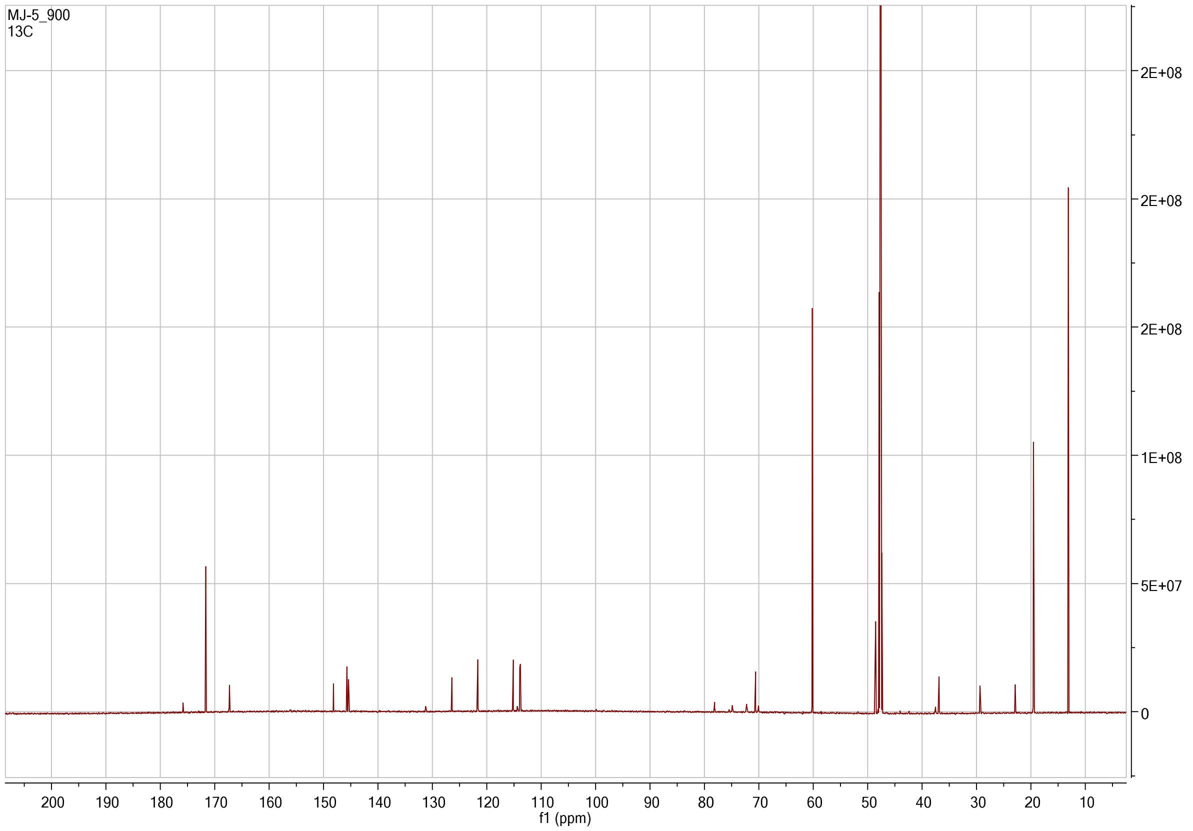

Figure S17. ^{13}C-NMR spectrum of compound **4** ($CDCl_3$ 225 MHz).

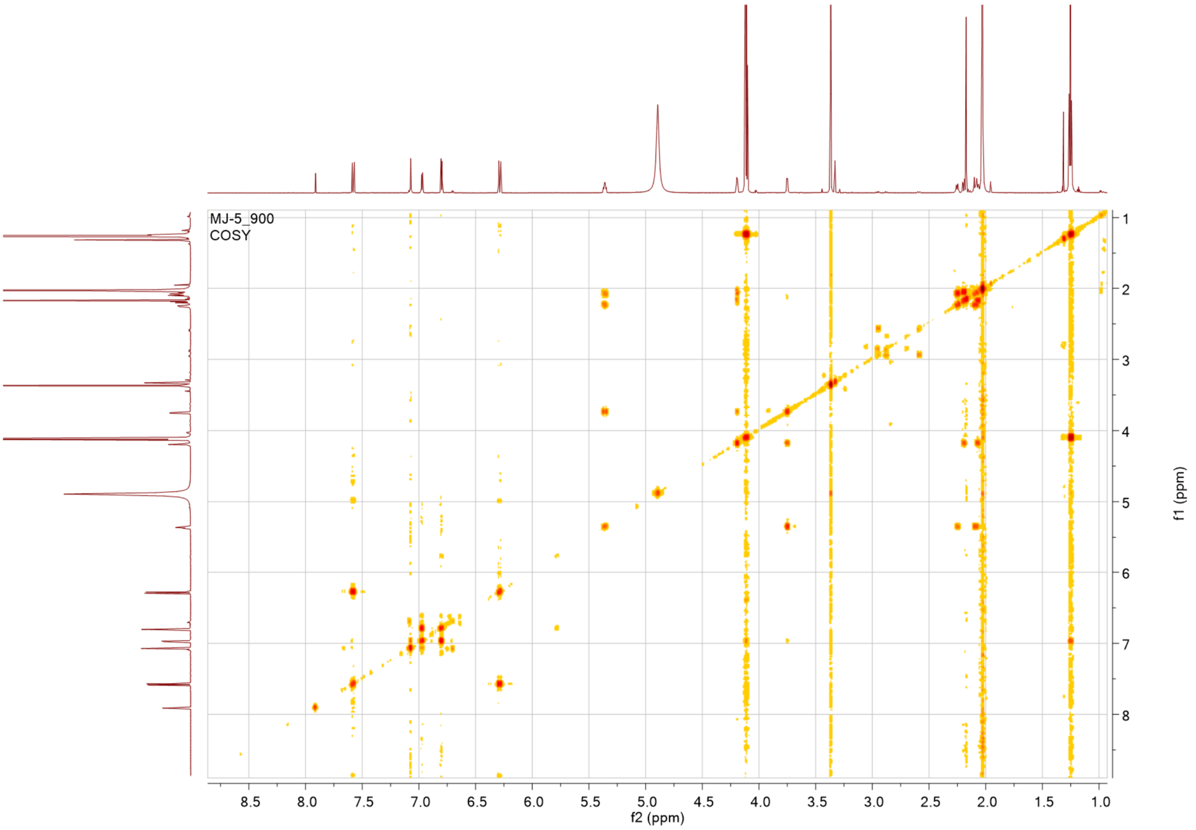

Figure S18. ^{1}H-^{1}H COSY spectrum of compound **4**.

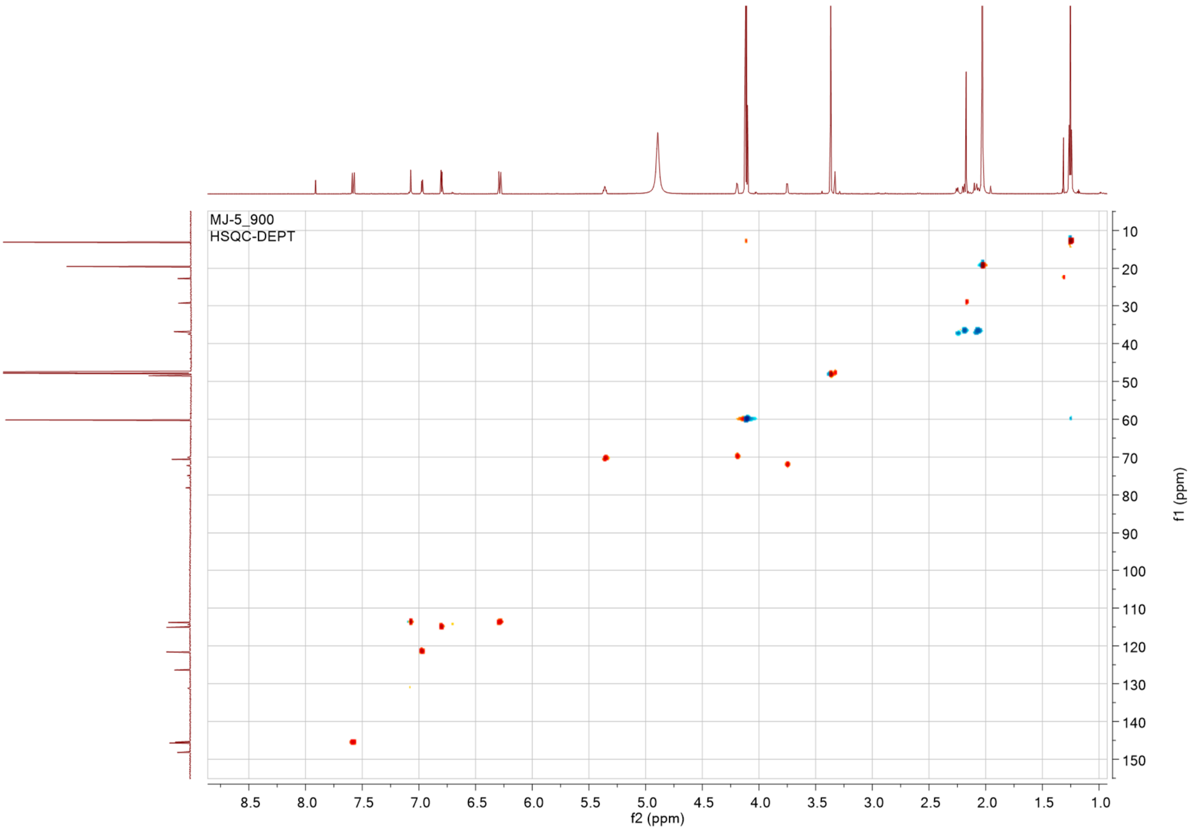

Figure S19. HSQC-DEPT spectrum of compound **4**.

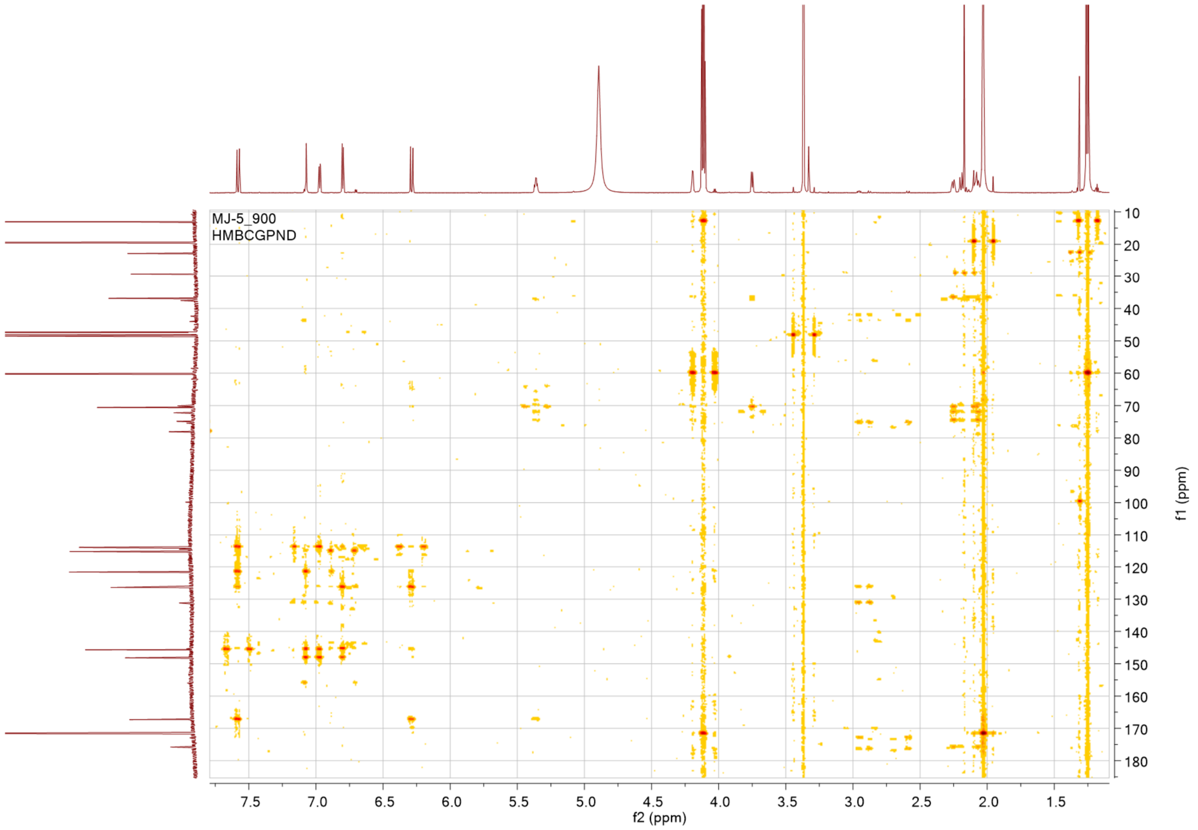

Figure S20. HMBC spectrum of compound **4**.

*5-O-Caffeoylquinic acid (**4**)*

^{1}H-NMR (900 MHz, Methanol-d_4) 7.58 (1H, d, *J* = 15.9 Hz, H-7′), 7.07 (1H, d, *J* = 1.9 Hz, H-2′), 6.97 (2H, dd, *J* = 8.2, 1.8 Hz, H-6′), 6.80 (1H, d, *J* = 8.1 Hz, H-5′), 6.29 (1H, d, *J* = 15.9 Hz, H-8′), 5.36 (1H, m, H-5), 4.20 (1H, m, H-4), 3.75 (1H, dd, *J* = 8.6, 3.0 Hz, H-3), 2.18 (1H, m, H-6), 2.18 (1H, m, H-2), 2.08 (1H, m, H-2), 2.07 (1H, m, H-6). ^{13}C-NMR (225 MHz, Methanol-d_4) 175.7 (C-7), 167.2 (C-9′), 148.2 (C-4′), 145.6 (C-7′), 145.4 (C-3′), 126.4 (C-1′), 121.6 (C-6′), 115.1 (C-5′), 113.9 (C-8′), 113.8 (C-2′), 74.9 (C-1), 72.3 (C-4), 70.6 (C-3), 70.1 (C-5), 37.6 (C-2), 36.9 (C-6).

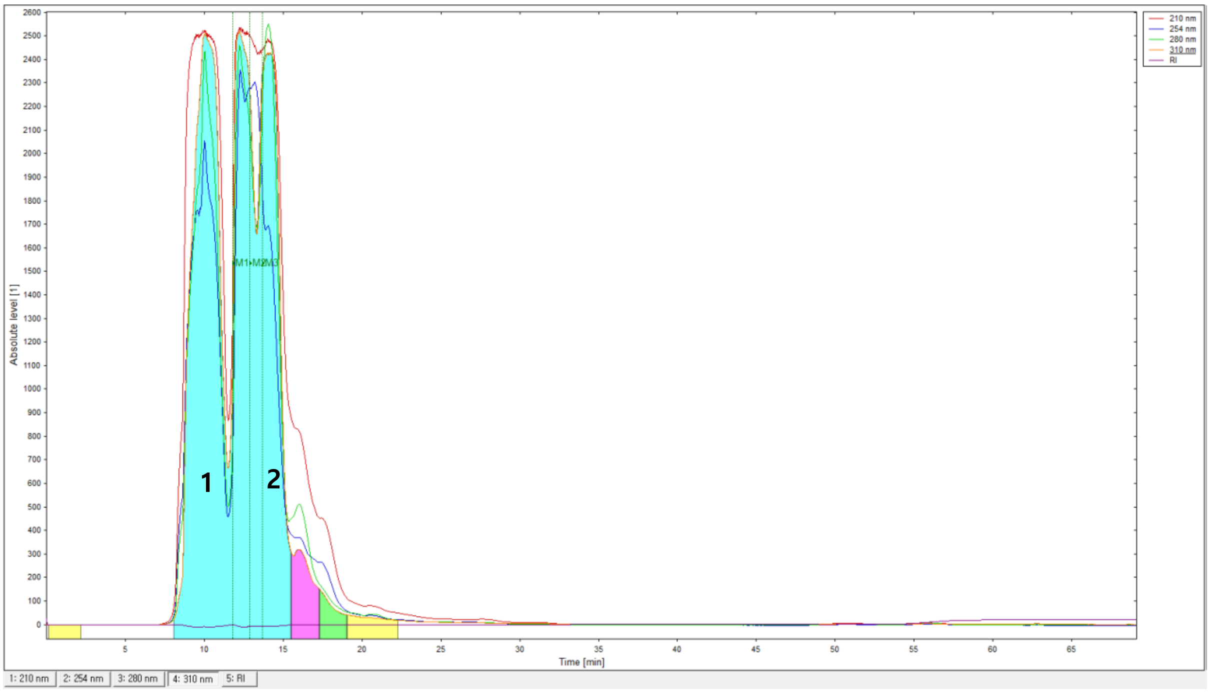

Figure S21. Pattern of purified substance from bakkenolides (compound **1** and **2**) of PB3 on preparative HPLC. The fractions were detected by UV (210, 254, 280, and 310 nm) and RI detector.

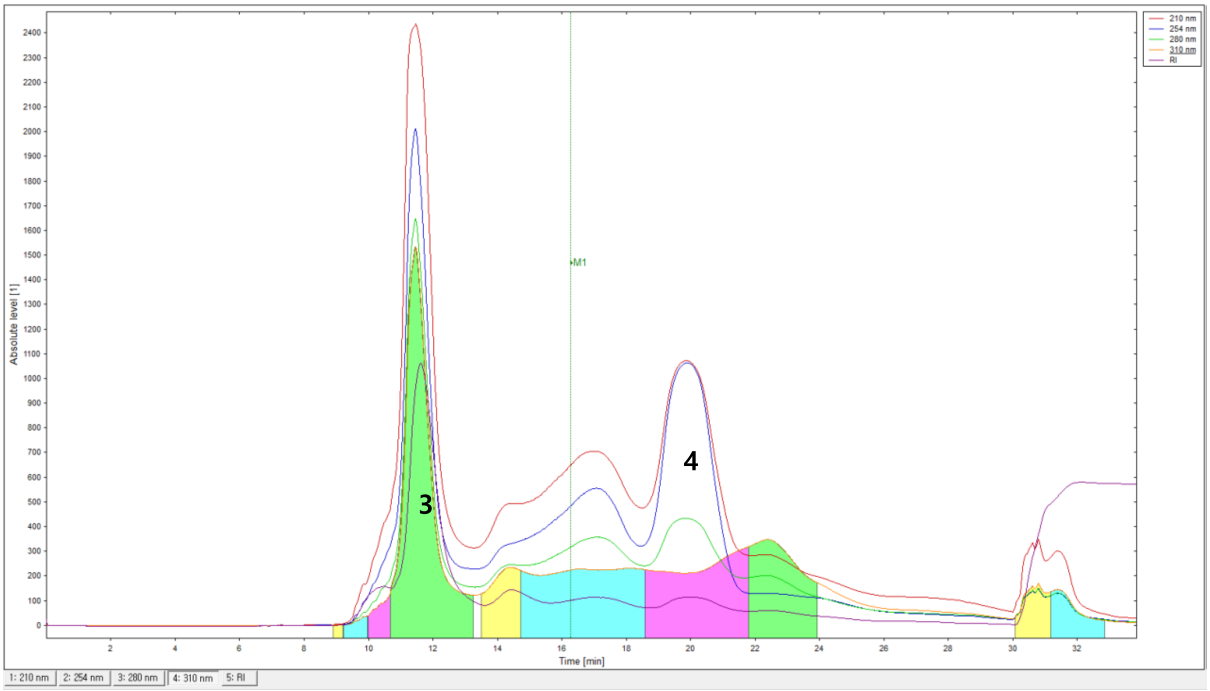

Figure S22. Pattern of purified substance from caffeoylquinic acids (compound **3** and **4**) of PB5 on preparative HPLC. The fractions were detected by UV (210, 254, 280, and 310 nm) and RI detector.

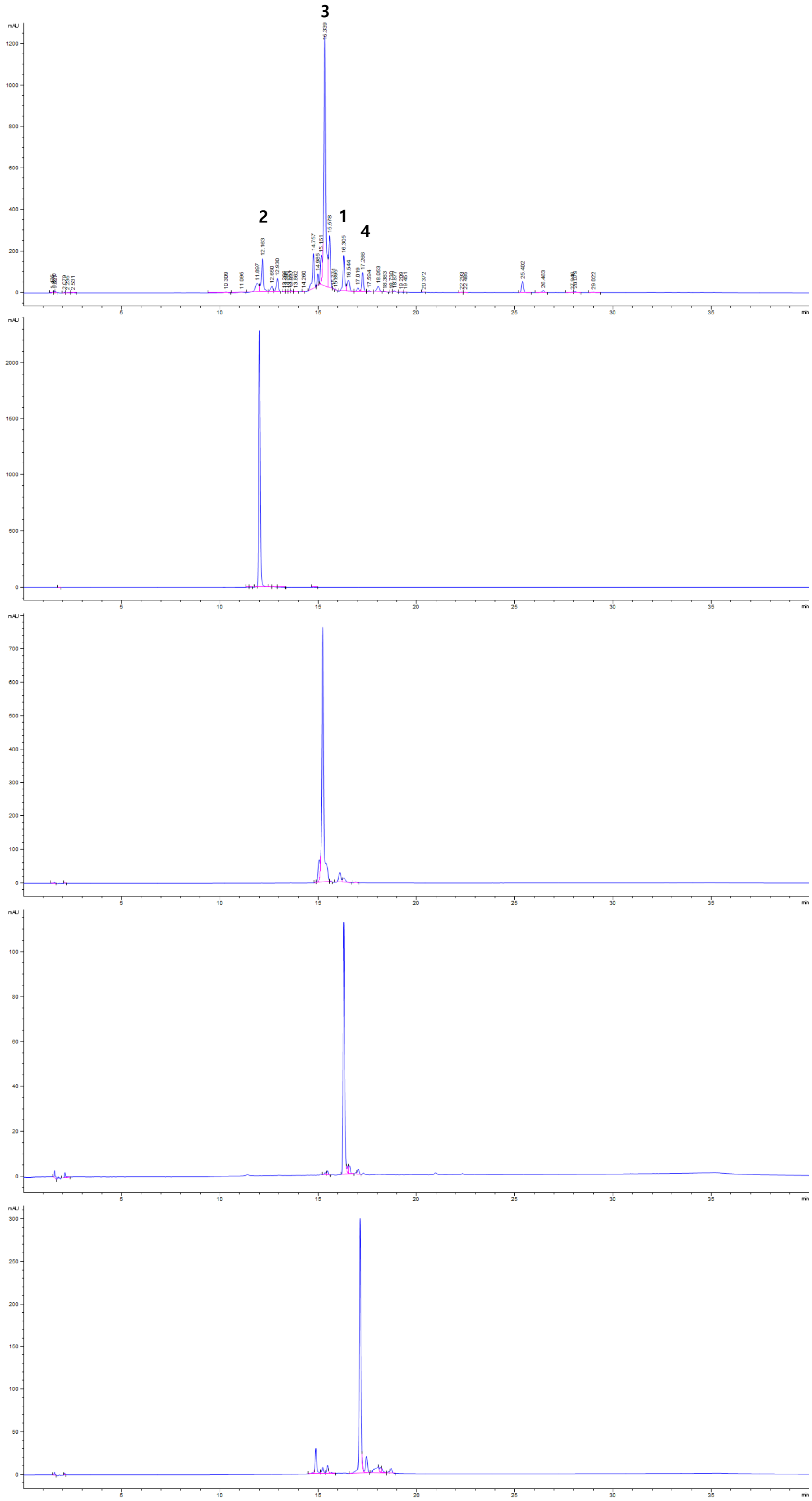

Figure S23. Methanol crude extract and compounds (**1**–**4**) from the aerial portion of *P. japonicus* were analyzed using a RP-18 HPLC column and detected with UV detector at 310 nm.

.

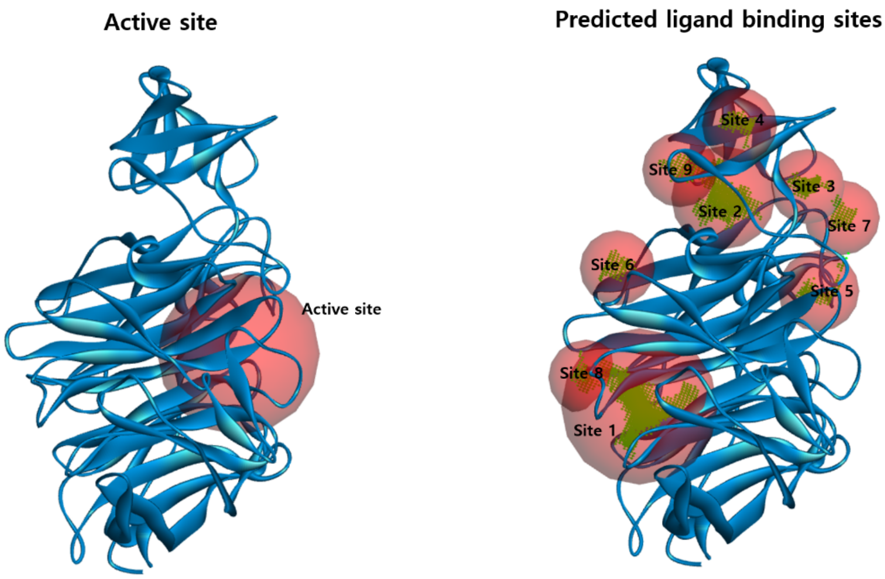

Figure S24. Active site and predicted ligand binding sites of neuraminidase. Red spheres represent cavities in which the ligand can be docked.

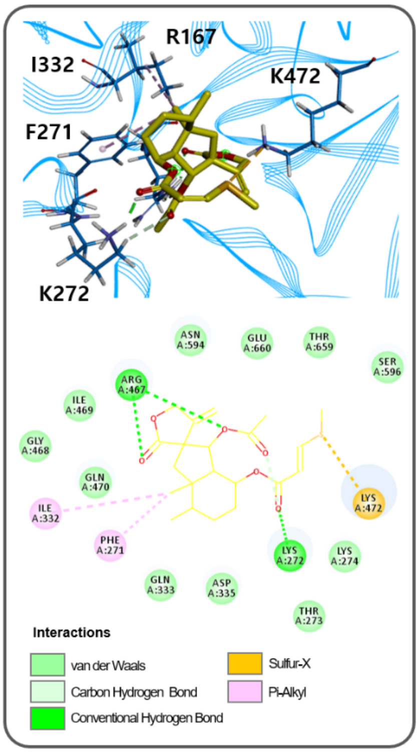

Figure S25. Docking pose of bakkenolide D. 3D- and 2D-structures represent receptor-ligand interaction. Bakkenolide D was represented as yellow stick models.

Table S1. C-DOCKER interaction energy and binding energy of docking poses at predicted ligand binding sites.

Pose	Site 1		Site 2		Site 8	
	C-DOCKER Interaction Energy (kcal mol^{-1})	Binding Energy (kcal mol^{-1})	C-DOCKER Interaction Energy (kcal mol^{-1})	Binding Energy (kcal mol^{-1})	C-DOCKER Interaction Energy (kcal mol^{-1})	Binding Energy (kcal mol^{-1})
1	−49.340	−69.603	−47.676	−49.772	−27.728	−35.507
2	−48.787	−62.790	−46.594	−48.940	−27.486	−32.543
3	−49.448	−64.977	−47.446	−56.250	−28.104	−33.309
4	−48.935	−74.346	−42.835	−61.653	−25.943	−20.786
5	−41.526	−71.955	−42.593	−43.141	−27.370	−29.821
6	−47.706	−71.269	−42.482	−70.948	−27.444	−39.227
7	−42.280	−71.460	−42.222	−67.113	−26.904	−27.708
8	−41.296	−74.407	−46.864	−32.758	−27.440	−40.170
9	−42.716	−78.460	−45.737	−16.861	−27.868	−39.431
10	−44.790	−71.720	−42.288	−34.620	−26.409	−34.657
